# Metastatic Melanoma to the Orbit With Dedifferentiation: A Case Report

**DOI:** 10.7759/cureus.41591

**Published:** 2023-07-09

**Authors:** Harkaran S Rana, Jake E Dertinger, Carson Clabeaux, Nicole Makepeace, Jason Lewis

**Affiliations:** 1 Ophthalmology, Walter Reed National Military Medical Center, Bethesda, USA; 2 Ophthalmology, California Health Sciences University College of Osteopathic Medicine, Clovis, USA; 3 Ophthalmology, Madigan Army Medical Center, Bethesda, USA

**Keywords:** orbital metastases, palliative debulking of tumor, perineurial spread, orbital tumor, intraorbital mass, metastatic melanoma

## Abstract

We present the first documented case of metastatic melanoma to the orbit with dedifferentiation. A patient with a history of melanoma of the lip and other poorly differentiated carcinomas presented with both a sub-brow and an intraorbital mass. Radiographically and intraoperatively, the sub-brow mass communicated with the intraorbital mass via perineural spread along the supraorbital nerve. Histopathology confirmed the diagnosis of melanoma based on the melanocytic markers, SOX-10 and Melan-A; dedifferentiation was demonstrated within the orbital tumor. Two weeks following surgical debulking, the intraorbital mass returned to its full size. Local radiotherapy and immunotherapy were performed, which initially led to a dramatic improvement; however, the patient succumbed to his systemic metastases six months later.

Dedifferentiation serves as a prognostic indicator and should be considered in patients when histopathology does not lead to a definitive diagnosis.

## Introduction

Common markers for melanoma aid in both the diagnosis and treatment of this malignancy; however, dedifferentiation presents a challenge for histologic diagnosis and imparts a poor prognosis for these patients [1-2]. In this report, we discuss the first reported case of dedifferentiation in metastatic orbital melanoma and highlight the aggressive nature of this tumor. This case report emphasizes the importance of thorough history-taking when encountering patients with prior poorly differentiated carcinomas.

## Case presentation

A 64-year-old Caucasian male presented to the emergency department at an outside hospital with restricted extraocular movement (EOM), decreased vision, and pain in the right eye (OD). The left eye (OS) was unaffected. The patient had an extensive oncologic history, comprising poorly differentiated parotid gland carcinoma, melanoma of the lip and neck, and a poorly differentiated carcinoma involving the left mandible and Meckel’s cave. The physical exam showed a firm sub-brow mass superolateral to the supraorbital notch, proptosis, and significant right upper eyelid edema with ptosis. CT scan demonstrated two enhancing lesions of the right orbit: a mass in the pre-septal tissue of the upper eyelid measuring 1.4 x 0.9 x 1.3 cm, and another intraorbital mass in the intraconal and extraconal spaces measuring 2.2 x 1.7 x 1.8 cm.

**Figure 1:**
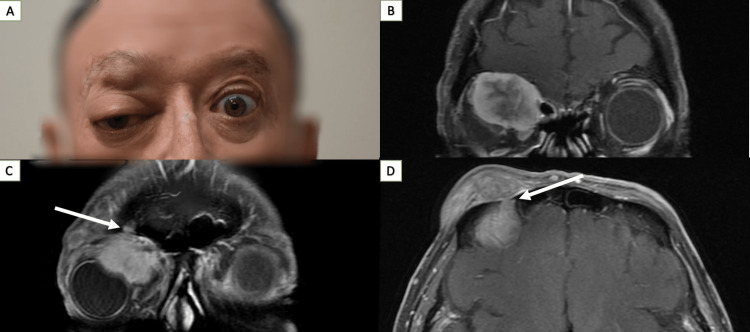
A: Patient with proptosis OD. B-D: MRI of pre-septal mass and intraorbital mass

The patient presented to our clinic 21 days after his initial visit to the outside facility. Best-corrected visual acuity (BCVA) was 20/50 OD and 20/20 OS. EOM was less than 30 degrees in all fields of gaze, and Hertel exophthalmometry revealed 16 millimeters of proptosis OD (Figure 1A). Color vision was normal for both eyes. Slit-lamp exam revealed exposure keratopathy and macular folds OD. MRI revealed substantial interval enlargement of both masses relative to initial imaging. The pre-septal mass measured 1.2 x 3.5 x 1.2 cm, and the intraorbital mass measured 2.6 x 3.2 x 3.5 cm. Perineural spread along the supraorbital nerve was appreciated (Figure 1B-1D).

**Figure 2:**
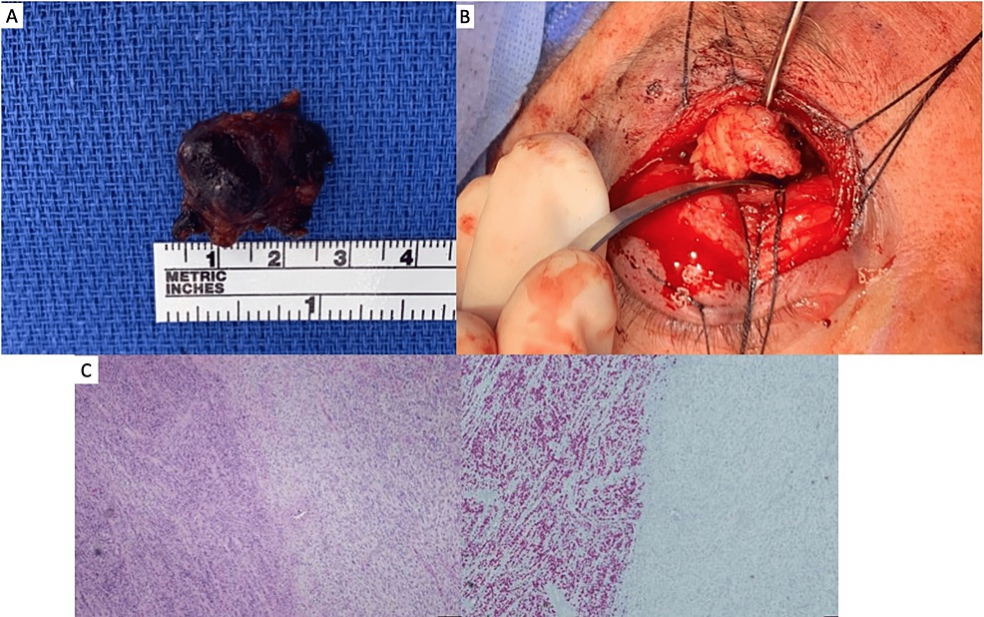
A: Sub-brow masses containing malignant tissue. B: Intraorbital tumor and supraorbital neurovascular bundle seen on dissection. C: Histology of both the sub-brow and intraorbital masses demonstrating distinct areas of loss of SOX-10 and Melan-A expression

Intraoperatively, we noted two distinct sub-brow masses connected by a bridge of malignant tissue, with projections interdigitating with the surrounding soft tissue. The masses were firm to palpation, black in color, and poorly vascularized relative to the surrounding soft tissue (Figure 2A). We continued dissection around the medial sub-brow mass, revealing that the mass was connected to the supraorbital neurovascular bundle. This was contiguous with the intraorbital tumor, which appeared tan/yellow in color, avascular, and lobulated (Figure 2B). Complete excision could not be performed, given the extent of soft tissue invasion and associated risk of optic nerve injury. Hence, the decision was made to debulk the intraorbital mass. Sufficient debulking was achieved once the proptosis had improved and the pressure to retropulsion was nearly symmetric. On postoperative day one, visual acuity (20/25 OD), proptosis, and EOMs were found to have markedly improved. Postoperative MRI demonstrated decreased mass effect on the globe. Histology of both the sub-brow and intraorbital masses demonstrated distinct areas of loss of SOX-10 and Melan-A expression, consistent with melanoma with dedifferentiation (Figure 2C). Areas with loss of SOX10 expressed a more spindled morphology with larger vesicular nuclei and prominent nucleoli that corresponded with a diagnosis of melanoma. Areas with SOX10 expression had hyperchromatic nuclei without conspicuous nucleoli and a more nested architecture consistent with melanoma.

Twelve days later, the patient started to experience vertigo, worsening proptosis, decreased vision, nausea, and confusion and presented to his local emergency department. A CT scan was ordered to rule out delayed intraorbital hemorrhage. Imaging revealed a 3.4 x 3 x 2.5 cm-sized mass, indicating aggressive characteristics of this malignancy. Further surgical management was deferred, and care was turned over to the patient’s radiation and medical oncologist. Local radiotherapy was performed, followed by monoclonal antibody therapy with pembrolizumab (Keytruda). Two months postoperatively, it was found that the patient had no light perception OD and there was complete paralysis of the right levator palpebrae secondary to his radiotherapy. Hertel exophthalmometry demonstrated 1 mm of proptosis. Despite an initial positive response to immunotherapy, the patient died at six months postoperatively while off immunotherapy due to systemic metastases to the liver and brain.

## Discussion

Our patient presented with metastatic dedifferentiated melanoma and perineural spread. We believe that the orbital mass represents a late metastasis from the patient's prior lip and neck melanoma. Additionally, the history of poorly differentiated carcinoma may have represented an undifferentiated lesion. Dedifferentiation refers to the process where a carcinoma loses features of its tissue of origin; an undifferentiated carcinoma has no morphologic similarity to its normal tissue counterpart [3]. Accurate histologic diagnosis is challenging if a dedifferentiated section is all that is sampled, and specific immunohistologic markers that aid in diagnosis are lost. In the context of melanoma, these include S100, HMB-45, Melan-A, microphthalmia-associated transcription factor (MITF), and SOX-10.

The mechanism by which melanocytes lose these markers has been described as “phenotypic plasticity” or “phenotypic switching,” in which gene expression and, therefore, the phenotype is influenced by the cellular microenvironment [1,2]. Multiple alterations in gene expression have been described in the literature. MITF and AXL are examples of phenotypic switching that can lead to a higher-grade neoplasm, often resistant to targeted therapy and immunotherapy [1]. If overly expressed, transcription factor MITF enables the development of a highly proliferative and poorly invasive melanoma. Decreased expression, seen in dedifferentiated melanoma, creates a slowly proliferative and highly invasive melanoma [2]. AXL is a tyrosine kinase, and its increased expression has been associated with promoting tumor growth and poor response to targeted treatment, resulting in a poor prognosis. Poor response to treatment in dedifferentiated tumors is extensively found in the literature [4,5,6]. A study by Müller et al. has described increased resistance to BRAF and ERK inhibition therapies in dedifferentiated MITF-low/AXL-high melanoma cells. The data suggested that MITF loss is a central determinant in receptor tyrosine kinase expression, particularly AXL, and drug resistance in melanoma [6].

While primary melanomas are most commonly cutaneous lesions, they can arise in the eye, orbit, and within mucous membranes. Melanoma can metastasize hematogenously, lymphatically, and, in rare cases, perineurally [7-9]. In a retrospective study of 89 patients with secondary orbital melanoma, the most common sites of primary origin of metastatic melanoma to the orbit were the choroid (51%) and conjunctiva (17%) [10]. Metastatic cutaneous melanoma accounts for 3-20% of metastases to the orbit and 1.4% of space-occupying lesions of the orbit in general [10-15].

Orbital metastases have appeared up to 34 years after diagnosing a patient's primary lesion [10]. In a systematic review of 27 patients with extraocular malignant melanoma, the mean time-to-diagnosis was 66 months (range: 21-106 months) [11]. Hence, there should be a high index of suspicion for metastatic orbital melanoma in patients presenting with an orbital mass, even if the history of melanoma is remote. Additional symptoms and signs suggestive of orbital melanoma include painless or painful proptosis, blurred vision, diplopia, dysmotility, and blepharoptosis [12,16,17].

Perineural spread of melanoma into the orbit occurs less commonly than squamous or basal cell carcinoma; it is well-documented in the literature (Table 1) [9,13, 17-24]. In the present case, the perineural spread was appreciated on MRI imaging and intraoperatively with the involvement of the supraorbital neurovascular bundle. As reported by Chang et al., when diagnosed on imaging, “direct extension of the tumor along the perineural tissue planes that follow the course of a named nerve" is seen [8]. Perineural invasion, also called neurotropism, is a separate classification and involves direct invasion of the tumor into the endoneurium [25]. In a review of eight cases of malignant melanoma of the head and neck with perineural spread, all patients demonstrated thickening and/or enhancement of involved nerves on MRI [9]. MRI is the gold standard and most sensitive imaging modality for evaluating tumors with perineural spread. It can provide high-quality soft-tissue contrast of tumors in the head and neck. PET scans may play a role in monitoring disease activity following treatment [26,27].

**Table 1:** Literature review of metastatic melanoma to the orbit with perineural spread DMM: desmoplastic malignant melanoma

Previously published studies of metastatic melanoma to the orbit with perineural spread and associated findings											
Author, year	Type of paper	Primary lesion/history	Years to orbital involvement	Presenting symptom	Location of Mets	Nerve(s) involved	Presence of desmoplasia	Tumor markers	Radiology description	Intervention	
Shields et al., 1987 [17]	Case report	Nasal cutaneous melanoma	5.5 years	Proptosis, blurry vision	Orbital apex	NR	+	S100+	Irregular mass filling the left orbit superonasally and posteriorly, extending to the orbital apex. No bony erosion		Radiotherapy orbital exenteration with the removal of the medial canthal area and upper eyelid
Khalil and Duguid, 1987 [18]	Case report	Malignant melanoma	6 years	Bells palsy, parotid mass, right-sided neuralgia	Orbit, lacrimal gland	CN VII, CN V_1_ & V_2_	+	S100+	NR		Radical orbitectomy and Radiotherapy
Hufnagel et al., 1990 [19]	Case report	Spindle cell tumor and lentigo maligna of the forehead	26 years	Afferent pupillary defect, CN III & VI palsy, numbness in V_1_ & V_2_ distribution progressing to complete ophthalmoplegia with CN VII palsy and pain	Medial orbital wall and superior orbital fissure	CN III, CN IV, CN V_1 _& V_2_, CN VI, CN VII	NR	S-100+, HMB-45+	Orbital mass eroding the medial wall and extending through the superior orbital fissure		NR, the patient died 2 months after the diagnosis
Ellis et al., 1994 [20]	Case report	DMM of the forehead	1.5 years	Diplopia, decreased vision, and painful forehead lesions	NR	NR	+	S100+, HMB-45-	CT: subcutaneous lesion with extension into the left orbit		External beam radiation of 5 x 6 Gy for a total of 31 Gy
Chang et al., 2004 [9]	Retrospective case series of 8 patients	1/8: family history of melanoma; 3/8: lentigo maligna; 4/8: none		NR	NR	CN III: 3, CN IV: 2, CN V_1_: 4 CN V_2_: 4, CN V_3_: 5, CN VI: 4, CN VII: 4, CN VIII: 1, CN IX: 1, CN X: 1, CN XI: 1, CN XII: 1	5/8 +	NR	MRI: gross perineural spread of tumor in all patients		5/8: conventional external beam radiotherapy; 4/8: gamma knife radiosurgery; 2/8: chemotherapy
Turell and Char, 2007 [21]	Case report	Aceniform papule, malignant melanoma	6 years	Periocular pain	NR	Infraorbital nerve	NR	NR	MRI: infraorbital nerve involvement, CT: increase in infraorbital nerve size		Surgical resection of the orbital floor and infraorbital nerve, partial maxillectomy
Hashemi et al., 2009 [22]	Case report	Malignant melanoma	4 years from the excision of the primary lesion	Trigeminal neuralgia, diplopia, facial paresthesia	Lateral orbital wall	CN V_2_, CN V_3_	+	S100+	MRI: homogenous contrasting lesion within the Gasserian ganglion along the 2nd and 3rd branches of the trigeminal nerve		Surgical resection, chemotherapy, and radiation
Chua et al., 2017 [13]	Case report	Neurotropic melanoma of the scalp	3 years from the excision of the primary lesion	Ptosis, diplopia	NR	Supraorbital and frontal branches of CN V_1_	+	S100+, Meln-A-, HMB-45-, BRAF-	MRI: perineural invasion of the supraorbital nerve and retrograde extension to the cavernous sinus		Patient declined intervention
Karlin et al., 2021 [23]	Case report	Melanoma of the forehead	6 years	Diplopia and pain	Superior orbital mass	Left supraorbital nerve	NR	NR	MRI: left superior orbit mass extending along the supraorbital nerve to the cavernous sinus, with indistinct borders and fat stranding		Ipilimumab, systemic corticosteroids, and external beam radiotherapy
Pompanin et al., 2021 [24]	Case report	Lentigo maligna melanoma of lip and gingiva	NR	Facial pain and hypoesthesia of CN V_2 _& V_3_	NR	CN V_2 _&V_3_	NR	S100+, BRAFV600+, HMB-45+	MRI: thickening of Gasserian ganglion and trigeminal root, maxillary branch		Proton beam therapy

## Conclusions

We presented the first reported case of metastatic melanoma to the orbit with perineural spread and dedifferentiation demonstrated on radiology and histopathology, respectively. The adaptability of malignant melanocytes to the local microenvironment poses a diagnostic and therapeutic challenge. With the incidence of melanoma doubling in the past four decades, we recommend a thorough review of personal and family oncologic history in these patients. We also encourage a complete dermatologic exam if there is a history of melanoma, and MRI is recommended as the primary imaging modality if there is a concern for perineural spread.
